# Physiological and Biochemical Adaptations to High Altitude in Tibetan Frogs, *Nanorana parkeri*

**DOI:** 10.3389/fphys.2022.942037

**Published:** 2022-07-06

**Authors:** Yonggang Niu, Xuejing Zhang, Tisen Xu, Xiangyong Li, Haiying Zhang, Anran Wu, Kenneth B. Storey, Qiang Chen

**Affiliations:** ^1^ Department of Life Sciences, Dezhou University, Dezhou, China; ^2^ School of Life Sciences, Lanzhou University, Lanzhou, China; ^3^ Department of Biology, Carleton University, Ottawa, ON, Canada

**Keywords:** *Nanorana parkeri*, high-altitude, hematological parameters, oxidative stress, antioxidant defense

## Abstract

The Xizang plateau frog, *N. parkeri* (Anura: Dicroglossidae), is endemic to the Tibetan Plateau, ranging from 2,850 to 5,100 m above sea level. The present study explores physiological and biochemical adaptations to high altitude in this species with a particular emphasis on parameters of hematology, oxidative stress, and antioxidant defense in adult and juvenile *N. parkeri* collected from high (4,600 m a.s.l) and low (3,400 m a.s.l) altitudes. Hematological results showed that hemoglobin concentration ([Hb]), hematocrit (Hct), and red blood cell (RBC) counts were significantly higher in high-altitude *N. parkeri*. High-altitude juveniles had lower RBC sizes than low-altitude juveniles. Higher levels of GSH and GSSG were indicated only in juveniles from high altitude, not in adults. High-altitude individuals also showed lower oxidative damage, assessed as malondialdehyde (MDA) and carbonyl groups (CG) in the liver. High-altitude adults also showed higher activities of superoxide dismutase (SOD), catalase (CAT), glutathione peroxidase (GPX), and glutathione-S-transferase (GST) as well as total antioxidant capacity (T-AOC) in the liver as compared to low-altitude adults. Moreover, higher GPX activity and T-AOC were observed in the heart and brain of high-altitude adults. Liver CAT, GPX, and T-AOC showed significant increases in high-altitude juveniles. Vitamin C content was also higher in the heart of high-altitude frogs compared to low-altitude individuals. In summary, the high-altitude population of *N. parkeri* showed more robust hematological parameters, less oxidative damage, and stronger antioxidant defenses than the low-altitude population, all contributing to increased protection for survival in high-altitude environments.

## Introduction

Environmental factors, including temperature, humidity, atmospheric pressure, and light intensity, vary significantly with altitude (Körner, 2007). The Qinghai-Tibet Plateau is the highest plateau in the world (average elevation above 4,000 m) and its extreme environment provides an ideal natural laboratory for investigating adaptive evolution (Qiao et al., 2016). Although hypoxia, cold temperature, and intense ultraviolet radiation (UVR) associated with high altitude environments pose serious challenges to animal survival, native species inhabiting the highlands thrive and prosper. One typical species is the Xizang plateau frog, *N. parkeri* (Anura: Dicroglossidae) (Zhang et al., 2012), that ranges from 2,850 to 5,100 m above sea level (a.s.l) and is an excellent model for studying adaptations of ectothermic animals to extreme environments. Our previous studies of this frog species focused on the physiological ecology of winter hibernation (Niu et al., 2022) and showed that overwintering *N. parkeri* frogs exhibit a seasonal suppression of metabolism and can tolerate brief and partial freezing of the body (Niu et al., 2020; Niu et al., 2021a; Niu et al., 2021c). Moreover, overwintering *N. parkeri* showed a higher level of oxidative stress and lower antioxidant capacity than summer-active individuals (Niu et al., 2018). The whole genome of *N. parkeri* has been sequenced and provides genetic evidence to investigate the crucial evolutionary traits of this species (Sun et al., 2015). A previous report showed that over-represented gene ontology (GO) categories including blood circulation and response to hypoxia and UVR were related to adaptation to high altitude in the eastern matriline of *N. parkeri* (Wang et al., 2018). Compared to other low-altitude frogs, high-altitude *N. parkeri* had more epidermal capillaries and granular glands in the skin structure, which may be an adaptation to cold and/or UVR (Yang et al., 2019). However, there have been few investigations into the physiological and biochemical adaptations to high altitude in *N. parkeri*.

Many studies have reported that high-altitude ectotherms can adapt to this extreme environment through physiological and biochemical adjustments, such as reducing metabolic rate (Tang et al., 2013), protecting against UVR (Reguera et al., 2014a), increasing pulmocutaneous blood flow (Gamperl et al., 1999), reducing erythrocyte volume (Ruiz et al., 1983), improving blood oxygen affinity by increasing red blood cell counts (RBCs), hemoglobin concentration ([Hb]) and hematocrit (Hct) (Lu et al., 2015). For example, the high altitude (3,800 m) Lake Titicaca frog, *Telmatobius culeus*, had smaller blood cells and higher blood O_2_ affinity than sea level frogs (Hutchison et al., 1976). Erythrocyte length in high-altitude *Batrachuperus pinchonii* was also lower than that in low-altitude individuals, a feature that can improve blood circulation and gas exchange rates (Xiong et al., 2018). Oxygen carrying capacity can be enhanced by increased hemoglobin concentration and hemoglobin-oxygen affinity as well as by changing the shape of the O_2_ equilibrium curve (Brauner and Wang, 1997; Storz, 2016). For instance, high-altitude *Bufo spinulosus flavolineatus* show higher hemoglobin affinity for oxygen than low-altitude individuals (Ostojic et al., 2000).

Animals exposed to high altitude may also suffer from oxidative/reductive stress and oxidative damage due to enhanced generation of reactive oxygen species (ROS) that are inevitable by-products of aerobic respiration, and the severity of oxidative challenge is related to the degree of altitude (Dosek et al., 2007). If intracellular ROS production remains at high levels, redox homeostasis is disrupted and damage occurs since ROS can directly attack biological macromolecules such as lipids, proteins, and DNA (Sies, 1997; Ray et al., 2012). The products of oxidative damage mainly include lipid peroxides, carbonyl proteins, and 8-oxo-7,8-dihydro-2′-deoxyguanosine (8-oxodG) (Grundy and Storey, 1998). However, animals can utilize highly effective antioxidant defenses, and efficient repair and removal mechanisms to combat ROS and eradicate damaged macromolecules. Antioxidant enzymes, including superoxide dismutase (SOD), catalase (CAT), glutathione peroxidase (GPX), glutathione-S-transferase (GST), and glutathione reductase (GR), play important roles in preventing or counteracting oxidant accumulation. Non-enzymatic antioxidants mainly include glutathione (GSH), ascorbic acid (vitamin C), α-tocopherol (vitamin E), and uric acid (Joanisse and Storey, 1996; Birben et al., 2012). It is generally accepted that increased aerobic metabolism or high oxygen levels produce more ROS, leading to altered redox homeostasis and oxidative damage (Giraud-Billoud et al., 2019).

Studies on the effects of high altitude at the level of oxidative stress and the antioxidant system are gradually increasing, but the findings are contradictory. For example, high-altitude lizards, *Psammodromus algirus*, had lower oxidative stress levels than low-altitude individuals (Reguera et al., 2014b). Compared to low-altitude individuals, other high-altitude lizards, *Phrynocephalus vlangalii*, showed lower T-AOC in liver and muscle tissues and higher levels of MDA in muscle (Zhang et al., 2015). At a simulated altitude of 5,500 m, Mn-SOD and GPX activities in the liver decreased significantly in rats, suggesting that the liver may be susceptible to oxidative stress induced by high altitude (Nakanishi et al., 1995). After 4 weeks of exposure at an altitude of 4,000 m, rats showed increased Mn-SOD activity in skeletal muscle, but no significant changes in CuZn-SOD, catalase, and glutathione peroxidase activities, as well as lipid peroxidation levels (Radák et al., 1997). Moreover, intermittent exposure (12 h/day) to a simulated altitude of 4,000 m induced a significant increase in the level of lipid peroxidation in the skeletal muscle of rats (Radak et al., 1994). These inconsistent findings may result from differences in 1) long-term adaptation to high altitude (under natural conditions) versus short-term acclimation (simulated conditions); 2) intensity and mode (e.g., intermittent and continuous) of exposure to high altitude; 3) different experimental protocols for measuring oxidative damage and oxidative stress indicators; and/or 4) species and tissue specificity. Overall, to scavenge ROS, the antioxidant defense system plays an important role in protecting organisms from high-altitude stress. However, limited information exists concerning the physiological and biochemical adaptations to high-altitude environments in *N. parkeri*.

We hypothesized that *N. parkeri* have more robust hematological parameters and stronger antioxidant defenses to cope with environmental stress at high altitudes where their populations are well-established. To test this hypothesis, we compared the hematological parameters and levels of oxidative stress and antioxidant defense between high- and low-altitude frogs, *N. parkeri*, both juveniles and adults. Indicators of oxidative stress (glutathione status) and oxidative damage (MDA; carbonyl groups, CG) were evaluated in liver and skeletal muscle tissues. Antioxidant systems, including antioxidant enzymes (SOD, CAT, GPX, GST, GR), a low molecular weight antioxidant (vitamin C), and total antioxidant capacity (T-AOC), were assayed in the heart, brain, liver, and skeletal muscle. Understanding these physiological and biochemical adjustments increases the current knowledge about evolutionary adaptations to high altitude in ectothermic vertebrates.

## Materials and Methods

### Sample Collection

**FIGURE 1:**
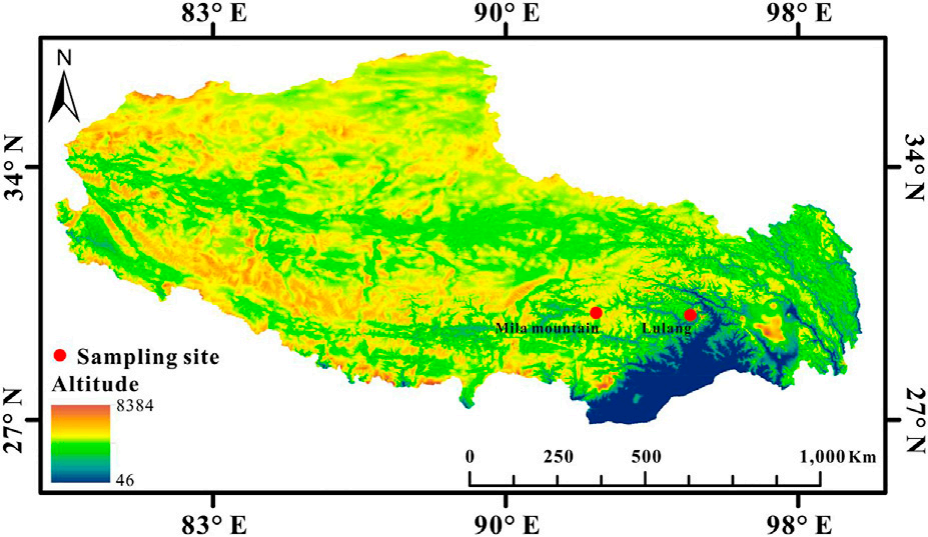
Sampling sites of high-altitude (4,600 m, Mila mountain) and low-altitude (3,400 m, Lulang) *N. parkeri* on the Qinghai-Tibet Plateau (Map source: http://westdc.westgis.ac.cn/).

*N. parkeri* adult males (*n* = 8 for each altitude) and juveniles (*n* = 16 for each altitude) were collected by hand from wetlands at 3,400 m (Lulang; 29.69°N, 94.73°E) and 4,600 m (Mila mountain; 29.75°N, 92.31°E) above sea level in July in Tibet, China (Figure 1). To eliminate the potential effects of body size on hematological parameters and oxidative stress levels, we selected juvenile and adult frogs with similar body sizes from the two altitudes. Body mass was weighed and snout-vent length was measured (Table 1), and then frogs were quickly euthanized by decapitation near the sampling site. Blood samples were collected from the aortic arch using heparinized glass capillary tubes. Fresh blood was immediately used to determine [Hb], Hct, and to make blood smears, with a portion of the fresh blood also diluted and transported to the laboratory for counting RBCs. Moreover, heart, brain, liver, and skeletal muscle were immediately removed and frozen in liquid nitrogen. Before biochemical analyses, liver and muscle samples (*n* = 8 for each group) from adults (but not juveniles) were divided into two parts. One portion of the tissue was used to determine glutathione status, and the remaining portion was used to test oxidative damage indicators and antioxidant enzyme activities. Due to the smaller hearts and brains of juveniles, eight samples were generated by combining tissue from two random individuals at the same altitude prior to biochemical analysis. All procedures were approved by the Ethics Committee of Animal Experiments at Lanzhou University and in accordance with guidelines from the China Council on Animal Care.

**TABLE 1:** Morphometric parameters of *N. parkeri* adults and juveniles collected from high- and low-altitude.

	Low-altitude adults	High-altitude adults	Low-altitude juveniles	High-altitude juveniles
Mean body mass (g)	4.45 ± 0.10	4.28 ± 0.10	2.61 ± 0.11	2.76 ± 0.07
Mean snout-vent length (cm)	4.01 ± 0.04	3.89 ± 0.06	3.21 ± 0.04	3.25 ± 0.03

The data are expressed as the means ± SEM (*n* = 8 for adults, *n* = 16 for juveniles, respectively).

### Hematological Parameters and Red Blood Cell Morphometrics

Hct was determined by centrifugation of whole blood for 10 min at 3,000 g in microhematocrit capillaries as described by He et al. (2013). Aliquots of 10 μl fresh blood were used to determine Hb concentration using a commercial kit (Nanjing Jiancheng Ltd. Co., China). RBCs counts were determined using a hemocytometer under a microscope. Mean corpuscular hemoglobin concentration (MCHC) was calculated as ([Hb]/Hct) × 100, mean corpuscular volume (MCV) was calculated as Hct/RBC count, and mean cell hemoglobin (MCH) was calculated as Hb/RBC count. Blood smears were stained using Wright-Giemsa stain (Beijing Solarbio Science & Technology Co., Ltd., China). We captured five images of the smear per animal using a fluorescence microscope (MDX6, Micro-shot Technology Co., Ltd., China). In each image, we measured the length (L, μm) and width (W, μm) of 10 erythrocytes, then the RBC size (μm^2^) was calculated based on the formulas LWπ/4. A total of 50 RBCs per animal and their mean value were used for statistical analysis. The measurements were performed using ImageJ analysis software (National Institutes of Health, United States).

### Preparation of Tissue Extracts

One portion of frozen liver or muscle (*n* = 8 for each group) was rapidly homogenized (1:5; w:v) in ice-cold (4°C) 5% (w:v) sulfosalicylic acid using an automatic low-temperature homogenizer (KZ-III-F; Wuhan Servicebio Technology Co., Ltd.). Homogenates were centrifuged at 4°C and 4,000 g for 10 min, and supernatants were collected for glutathione assay. In addition, other tissue samples (heart, brain, liver, and muscle) (*n* = 8 for each group) were weighed and immediately homogenized (1:9; w:v) in ice-cold sterile saline solution (0.65%). Homogenates were centrifuged at 4°C and 4,000 g for 10 min, and supernatants were collected for testing MDA and CG content and antioxidant capacity parameters. The supernatants were placed on ice and quickly used to determine all indicators within the same day.

### Biochemical Analyses

Principles of all biochemical analyses are shown in Supplementary File S1. All assays were conducted at 25°C ± 0.5°C using an automatic microplate reader (BioTek Instruments, Inc.) and commercial assay kits (Nanjing Jiancheng Ltd. Co., China). Each sample was measured twice, and the mean was used for statistical analyses. Protein concentration was assayed by the Bradford dye-binding method using bovine serum albumin as the standard (Van Kley and Hale, 1977).

### Statistical Analyses

All data were tested for normality and homogeneity of variances to meet the assumptions of parametric testing and presented as mean ± SEM. Student’s t-tests for independent samples were used to determine the differences between high and low altitudes. All statistical analyses were performed using SPSS 20.0 (SPSS, Inc., Chicago, IL, United States), and significance was accepted when *p* < 0.05.

## Results

### Hematological Parameters

Both adults and juveniles at high altitude showed higher values for RBC counts, [Hb], and Hct than low-altitude frogs (Table 2). By contrast, the value of MCHC was significant lower in high-altitude adults compared to low-altitude adults. For adults and juveniles from the same altitudinal site, the values of MCV and MCH showed no significant differences between the two altitudes (Table 2). The width and area values of erythrocytes in high-altitude juveniles were significantly lower than those in low-altitude juveniles, but no significant changes were found in adults (Table 2).

**TABLE 2:** Hematological parameters and red blood cell morphometrics of high- and low-altitude adult and juvenile *N. parkeri*.

	Low-altitude adults	High-altitude adults	Low-altitude juveniles	High-altitude juveniles
Hematological parameters				
RBC count (10^12^ L^−1^)	0.51 ± 0.01	0.62 ± 0.01***	0.46 ± 0.01	0.54 ± 0.01***
Hematocrit (%)	21.07 ± 1.03	28.16 ± 1.87**	22.51 ± 0.88	26.12 ± 1.09*
Hemoglobin (g L^−1^)	142.9 ± 7.19	169.2 ± 7.53*	112.9 ± 2.94	123.5 ± 2.23*
MCHC (g L^−1^)	568.5 ± 16.52	467.3 ± 14.2***	462.1 ± 11.97	477.4 ± 18.31
MCV (pL)	0.47 ± 0.03	0.47 ± 0.03	0.52 ± 0.03	0.47 ± 0.02
MCH (pg)	256.8 ± 14.22	236.5 ± 12.93	249.9 ± 9.80	232.2 ± 8.19
Red blood cell morphometrics				
Length (μm)	16.44 ± 0.41	16.54 ± 0.28	15.25 ± 0.19	15.33 ± 0.12
Width (μm)	12.25 ± 0.17	12.15 ± 0.12	11.85 ± 0.30	10.63 ± 0.12**
RBCs size (μm^2^)	158.1 ± 5.60	158.5 ± 3.77	143.3 ± 5.15	129.2 ± 2.39*

The data are expressed as the means ± SEM (*n* = 8). Student’s t-tests for independent samples were used to test for significant differences between the two altitudes (*p < 0.05, **p < 0.01, ***p < 0.001).

### Glutathione Status

**FIGURE 2:**
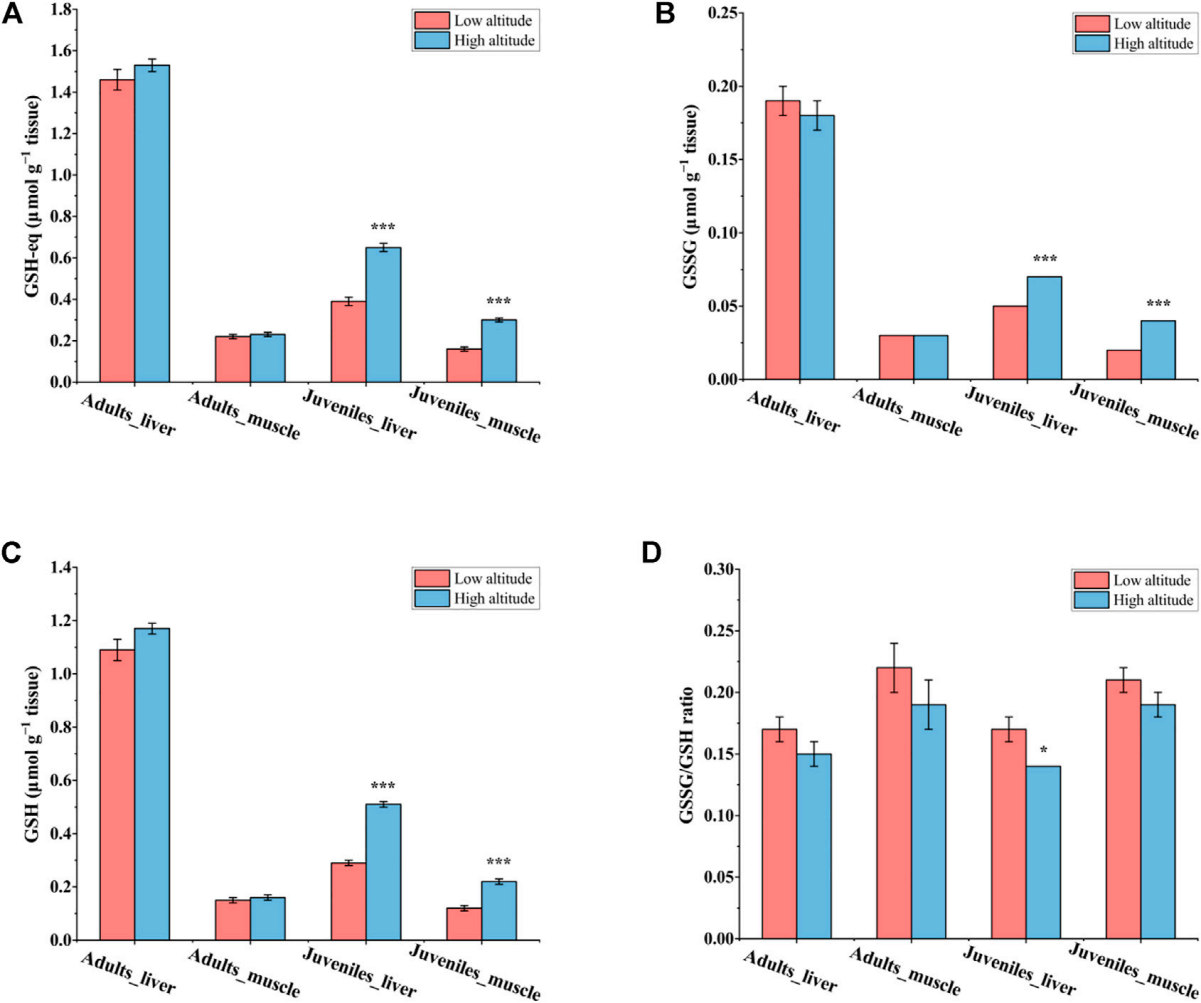
Glutathione status in the liver and skeletal muscle of *N. parkeri* adults and juveniles at low and high altitude, respectively. **(A)** GSH-eq, **(B)** GSSG, **(C)** GSH, **(D)** GSSG/GSH ratio. Total glutathione equivalents (GSH-eq) are GSH + 2GSSG. The data are expressed as the means ± SEM (*n* = 8) in μmol g^−1^ tissue. Student’s t-tests for independent samples were used to test for significant differences between the two altitudes (*p < 0.05, ***p < 0.001).

In liver and muscle, the content of GSH-eq, GSH, and GSSG as well as the GSSG/GSH ratio showed no significant differences between high- and low-altitude adult *N. parkeri* (Figure 2). However, in juveniles, the contents of GSH-eq, GSH, and GSSG were all significantly greater in both tissues of high-altitude frogs than in low-altitude individuals. Moreover, the GSSG/GSH ratio was significantly lower in liver of juveniles living at high altitude, as compared to low altitude (Figure 2).

### Oxidative Damage

**FIGURE 3:**
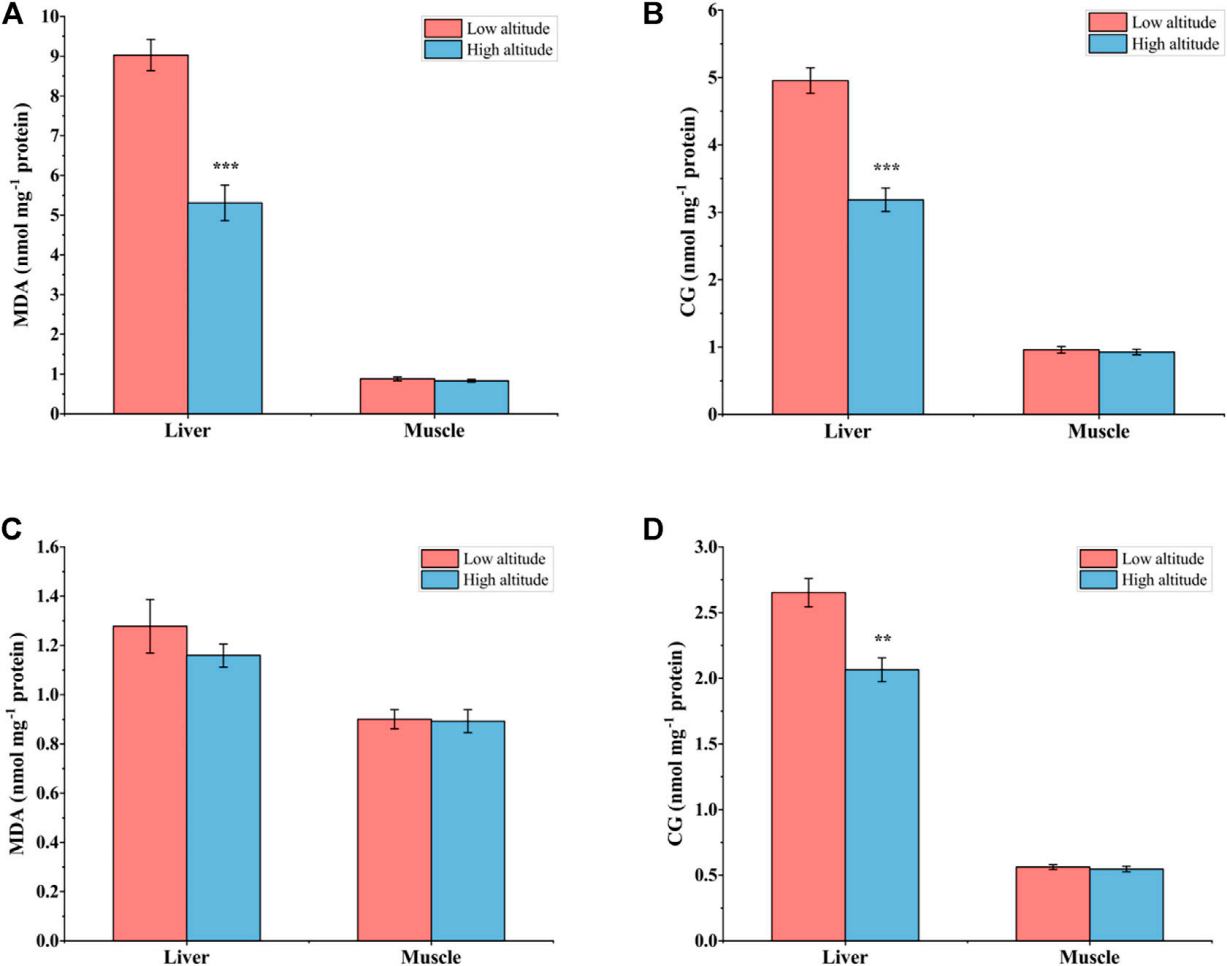
Malondialdehyde (MDA) **(A)** and carbonyl groups (CG) **(B)** in liver and muscle of low-altitude and high-altitude adult frogs, and comparable values for juvenile frogs **(C,D)**. Data are presented as mean ± SEM (*n* = 8). Asterisks (**p < 0.01, ***p < 0.001) indicate significant differences between two populations.

For adult frogs, MDA content in the liver was significantly lower (by ∼41%) in high-altitude frogs than low-altitude individuals, but no significant difference was found in muscle (Figure 3A). High-altitude frogs also had lower CG content (by ∼36%) in the liver relative to low-altitude individuals but, again, there was no significant difference in this parameter in muscle between the two altitudes (Figure 3B). For juvenile frogs, no significant differences in MDA content occurred in either liver or muscle tissues of *N. parkeri* (Figure 3C). CG content was lower by 22% in the liver of high-altitude frogs than in low-altitude individuals, but no difference was seen in muscle (Figure 3D).

### Antioxidant Defense Systems

**FIGURE 4:**
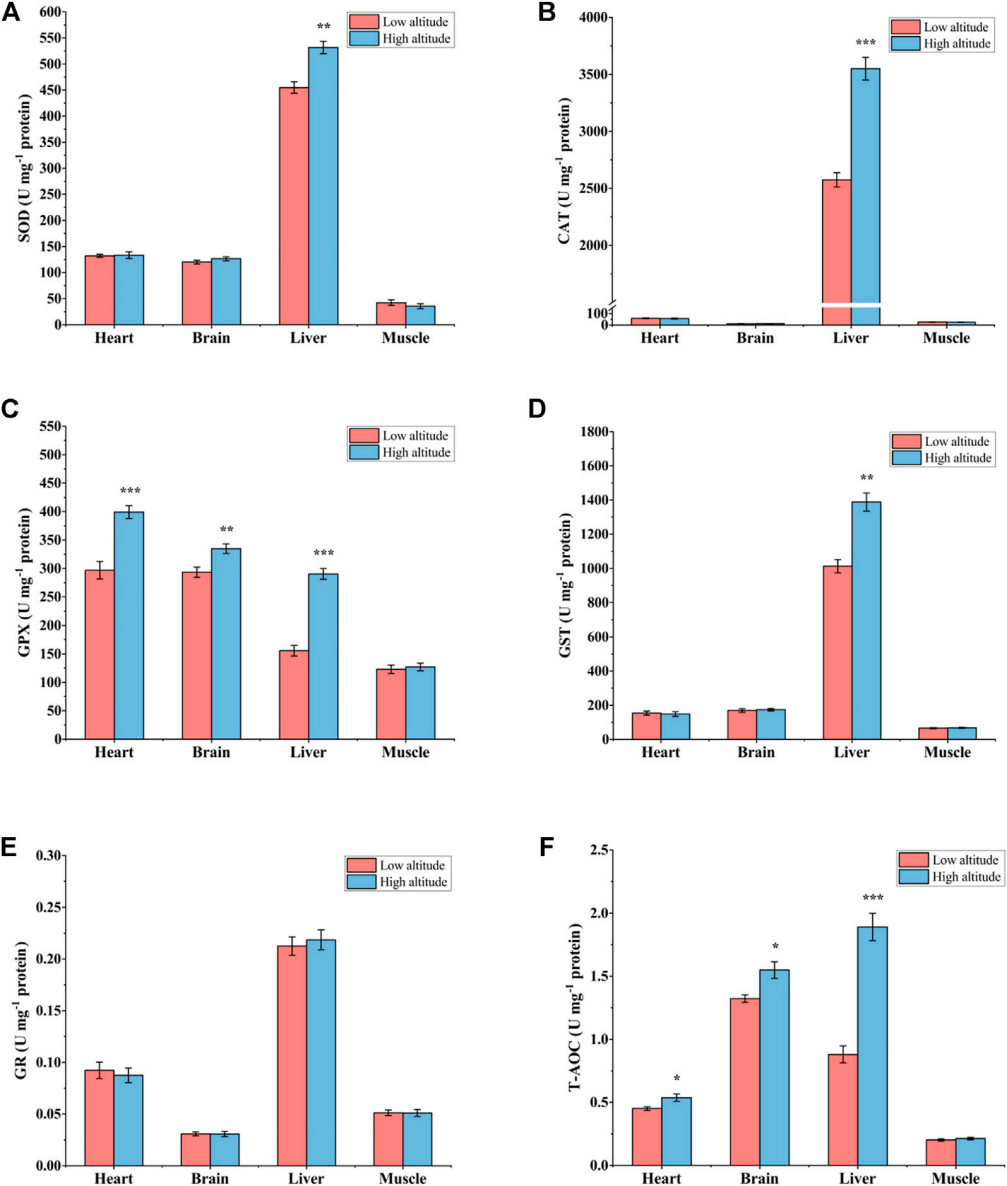
Antioxidant enzyme activity and total antioxidant capacity (T-AOC) in heart, brain, liver, and muscle of low-altitude and high-altitude adult frogs. **(A)** superoxide dismutase (SOD) activity, **(B)** catalase (CAT) activity, **(C)** glutathione peroxidase (GPX) activity, **(D)** glutathione-S-transferase (GST) activity, **(E)** glutathione reductase (GR) activity and **(F)** total antioxidant capacity (T-AOC). Values are presented as mean ± SEM (*n* = 8). Asterisks (*p < 0.05; **p < 0.01; ***p < 0.001) indicate significant difference between two populations.

For adult frogs, SOD activity was significantly higher (∼1.2-fold) in the liver of high-altitude frogs than in low-altitude individuals, but no significant differences were found in heart, brain or skeletal muscle (Figure 4A). Liver CAT activity was 38% greater in high-altitude frogs relative to low-altitude individuals, but CAT activity showed no significant difference in the other tissues examined (Figure 4B). GPX activity was significantly higher by ∼34% in heart, ∼14% in brain, and ∼87% in liver of high-altitude frogs, as compared with low-altitude individuals (Figure 4C). However, there were no significant differences in GPX activity in muscle between the two altitudes. GST activity was 1.4-fold higher in liver of high-altitude frogs than in low-altitude frogs, but no significant difference was observed in other tissues (Figure 4D). In all examined tissues, GR activity was unchanged between the two altitudes (Figure 4E). T-AOC exhibited a similar trend to that seen for GPX activity. Relative to low altitude frogs, T-AOC was significantly higher in high-altitude frogs by 20% in heart, 17% in brain, and 115% in liver. No significant difference was detected in muscle between the two altitudes (Figure 4F).

**FIGURE 5:**
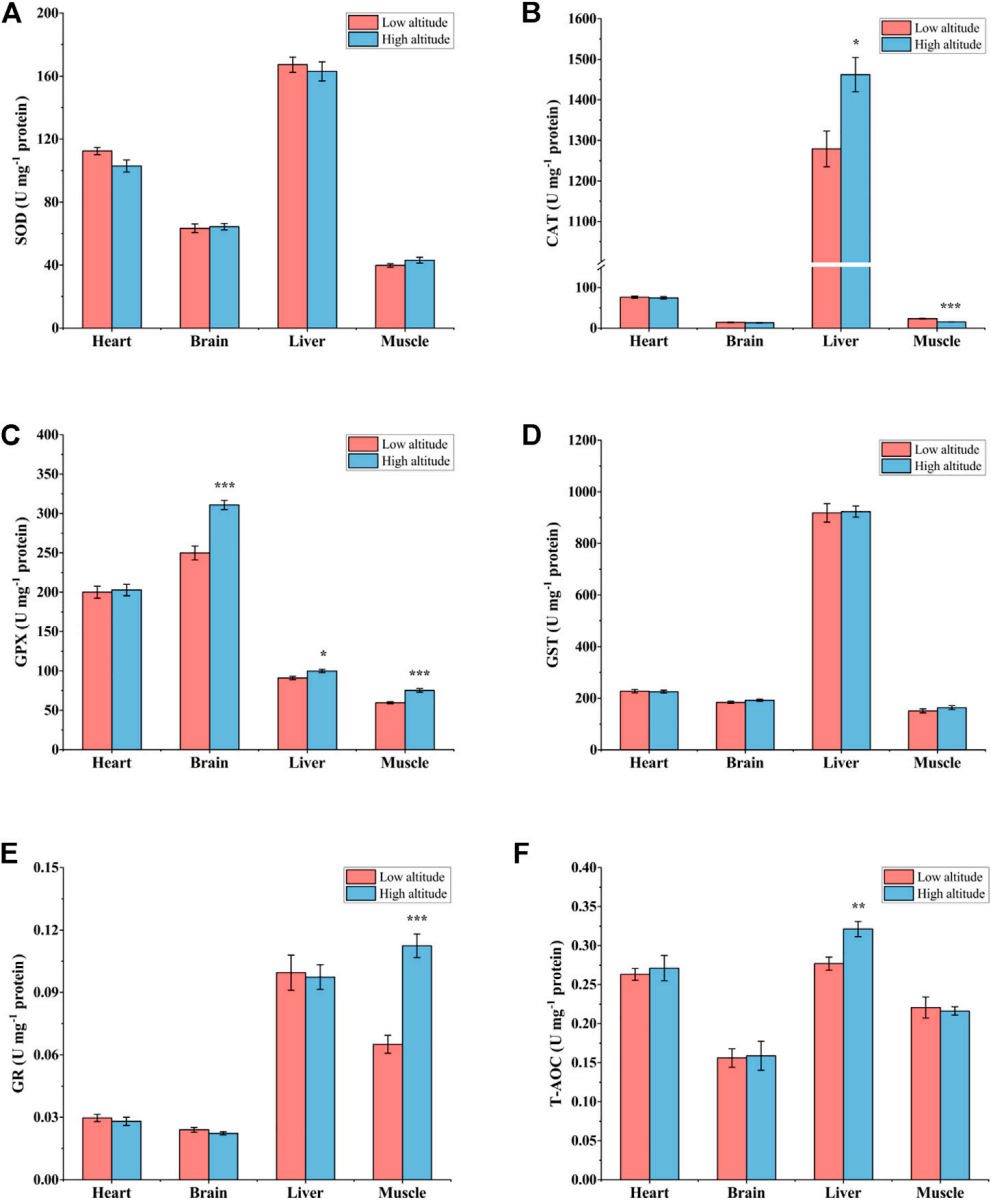
Antioxidant enzyme activity and total antioxidant capacity (T-AOC) in heart, brain, liver, and muscle of low-altitude and high-altitude juvenile frogs. **(A)** superoxide dismutase (SOD) activity, **(B)** catalase (CAT) activity, **(C)** glutathione peroxidase (GPX) activity, **(D)** glutathione-S-transferase (GST) activity, **(E)** glutathione reductase (GR) activity and **(F)** total antioxidant capacity (T-AOC). Values are presented as mean ± SEM (n = 8). Asterisks (*p < 0.05; **p < 0.01; ***p < 0.001) indicate significant difference between two populations.

For juvenile frogs, there was no significant difference in SOD activity and GST activity in all tested tissues between high- and low-altitude frogs (Figures 5A,D). However, compared to low-altitude frogs, CAT activity was 14% higher in liver but 33% lower in muscle in high-altitude individuals (Figure 5B). Except for the heart, GPX activity was significantly greater in all tissues of high-altitude frogs, by 24% in brain, ∼10% in liver, and 26% in muscle, respectively (Figure 5C). GR activity showed a 57% greater activity in muscle only as compared with high-altitude frogs, whereas no significant difference was observed in other tissues (Figure 5E). T-AOC was 14% higher in the liver of high-altitude frogs, but no significant difference was found in other tissues compared to low-altitude individuals (Figure 5F).

**FIGURE 6:**
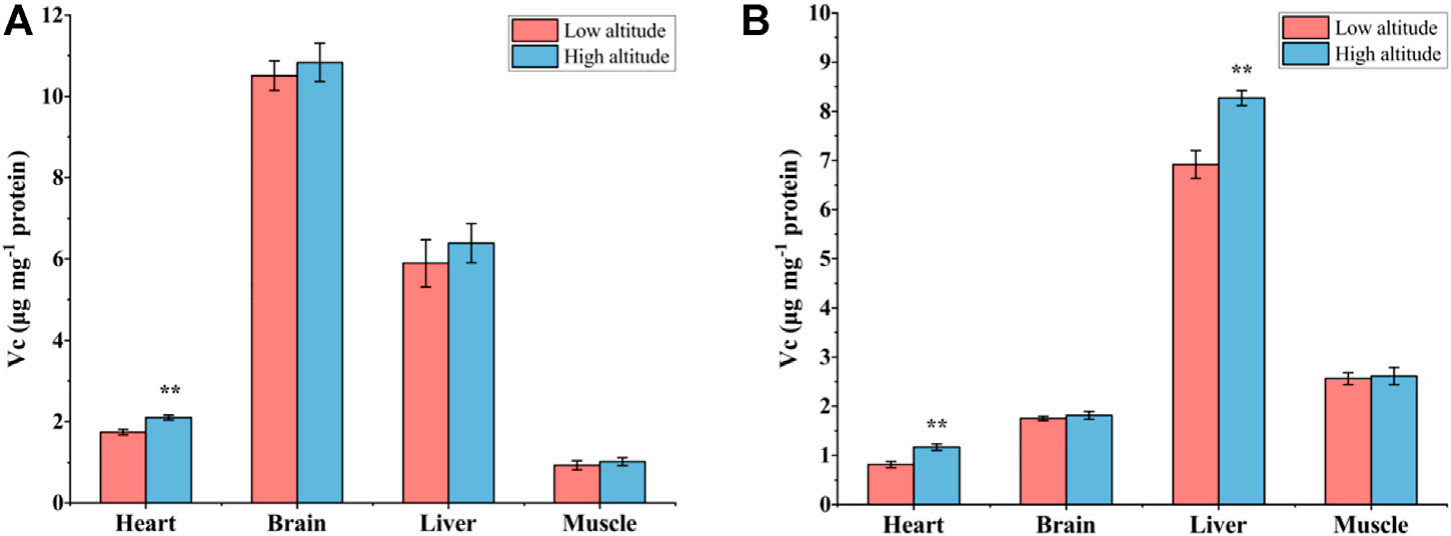
The contents of Vitamin C (Vc) in heart, brain, liver, and muscle of low-altitude and high-altitude adult **(A)** and **(B)** juvenile frogs. Values are presented as mean ± SEM (*n* = 8). Asterisks (**p < 0.01) indicate significant differences between two altitudes.

Vc content in the heart of high-altitude adults was significantly higher (1.2-fold) than that in low-altitude adults, with no significant differences observed in other tissues (Figure 6A). The Vc content of juvenile frogs at high altitude was also significantly higher by 20% and 44% in the liver and heart, respectively, compared to individuals at low altitude (Figure 6B).

## Discussion

Hematological parameters are an important marker that reveal the physiological adaptations of animals to their environment. Animals at high altitude typically increase the number of RBCs and their Hb concentration to improve oxygen-carrying capacity and adapt to the lower atmospheric oxygen levels at high altitude (Ruiz et al., 1983; He et al., 2013). Our study of high versus low altitude *N. parkeri* shows these same adaptations. A previous study showed that Hb levels in peripheral blood increased with altitude in the eastern lineage of *N. parkeri* (Wang et al., 2018), in agreement with our result that high-altitude frogs have higher Hb concentrations than low-altitude individuals. However, some amphibian and reptile species show different patterns of response to high altitude. For example, a previous study found no significant relationship between Hb concentration and altitude in chorus frogs (*Pseudacris triseriata*), possibly because the high altitude tested (3,000 m) was not an extreme altitude (Packard and Stiverson, 1976). The lizard, *Sceloporus grammicus*, showed no change in any blood trait with increasing altitude (González-Morales et al., 2017). Some species even show reverse responses to altitude as compared with *N. parkeri*. For example, the roughskin newt, *Taricha granulosa*, had lower Hb concentrations and RBC counts in the high altitude population than the lower altitude population (Friedmann, 1971). The toad, *B. spinulosus flavolineatus*, also had much lower hemoglobin concentration and smaller RBCs than two low-altitude subspecies (Ostojic et al., 2000). One potential reason for this may be that these species have adopted different adaptive strategies to cope with hypoxia, such as the differences in the allosteric regulation of Hb-O_2_ affinity (ATP, DPG) and pH. Furthermore, it has been widely demonstrated that significant hematological adaptations were observable only in anurans from extremely high elevations (e.g., >3000 m) (Ruiz et al., 1983).

It may seem harmful that high-altitude adult *N. parkeri* had higher Hct values than low-altitude adults, since higher Hct will increase blood viscosity and raise peripheral vascular resistance, thereby impeding circulation and O_2_ transport (Guyton and Richardson, 1961). However, Hct values in *N. parkeri* were actually lower than in other amphibians, such as *Rana catesbeiana*, where hematocrit is 40.4% ± 1.2% (Carmena-Suero et al., 1980). Therefore, elevation of Hct may not impair blood circulation processes in high-altitude *N. parkeri*. A similar finding was reported for the toad, *B. spinulosus*, where high-altitude populations (above 3,200 m) exhibited lower hematocrit values, smaller RBC size, and larger RBC counts than low-altitude toads (up to 2,700 m) (Ruiz et al., 1989). Smaller RBC size allows for higher gas exchange rates because the RBCs can readily pass through smaller and more densely distributed capillaries (Hutchison et al., 1976; Nikinmaa, 1997). The smaller RBC size at high altitudes has been widely reported in ectothermic vertebrates and is considered to be a physiological adaptation to the environment (Ruiz et al., 1983; Ruiz et al., 1989). Hematological parameters have also been reported to be different between age groups in amphibians (Allender and Fry, 2008), and our present study also showed that RBC counts and [Hb] were higher in adults than those in juveniles at the same altitude. Overall, these hematological characteristics may contribute to the successful life of *N. parkeri* at high altitudes.

It is generally assumed that high-altitude environments are characterized by hypoxia, low temperature, and strong UVR. These abiotic stresses can significantly affect ROS levels in organisms, either directly or indirectly, resulting in an oxidative stress state (Körner, 2007; Sola et al., 2008; Gaur et al., 2021). However, our present results suggest that high-altitude environments did not induce oxidative stress in this species. Similar results have been found in the lizard, *P. algirus*, where oxidative stress decreased with elevation (Reguera et al., 2014b). Higher GSH-eq and GSH contents in high-altitude juveniles may be related to greater synthesis of glutathione at high altitude. This result concurs with a previous study showing that hypoxia exposure enhanced GSH concentration by increasing *de novo* biosynthesis (D’Alessandro et al., 2016). GSH not only directly neutralizes ROS but also plays an important role as a cofactor for various glutathione-dependent antioxidant enzymes (Ribas et al., 2014). Thus, increased GSH content contributes to enhancing antioxidant capacity in high-altitude individuals. In the present study, a decrease in oxidative stress is evidenced by the lower levels of oxidative damage markers, such as MDA and CG.

Oxidative stress arises from an imbalance between pro-oxidants and antioxidants; therefore, one of the reasons for low levels of oxidative stress may be that there are fewer pro-oxidants in the high-altitude environment. The values of PO_2_ at the high (4,600 m) and low-altitude (3,400 m) sites in this study were 11.9 and 14.0 kPa, respectively, as compared with PO_2_ at sea level that is 21.2 kPa. Therefore, differences in oxidative stress levels between high- and low-altitude populations can also be attributed to the different PO_2_ level in their respective habitats. However, the metabolic rate of ectotherms will increase with rising ambient temperature, which can intensify the body’s oxygen consumption and lead to enhanced ROS production (Lushchak and Bagnyukova, 2006). On the contrary, low temperature would significantly decrease aerobic metabolic processes and slow down the rate of biochemical reactions, which could help to reduce the production of ROS and the risk of oxidative stress. In our study, the ambient temperature around the high-altitude frogs (13.67°C ± 0.33°C) was significantly lower than the value at the lower altitude (19.5°C ± 0.42°C). As altitude increases, UVR also rises significantly, which is one of the important factors that trigger oxidative stress (Dahms and Lee, 2010). Previous histomorphological results showed that skin structures in high-altitude *N. parkeri* contained more glands and pigmentation than low altitude individuals (Yang et al., 2019), which can effectively protect against UVR.

As altitude increases, environmental stress becomes more severe, including low temperatures and food shortages, which results in higher altitude frogs having more extended hibernation periods and shorter activity periods (Ma et al., 2009). Hence, high-altitude frogs experience more feeding restrictions, including shorter seasons when food (typically insects) is available. Indeed, food availability plays a crucial role in developing and maintaining all physiological functions (Metcalfe and Monaghan, 2001). Reduced food availability may induce changes in metabolic and endocrine function, affect enzyme activity and gene expression, and even help reduce the oxidative damage (Noguera et al., 2011). This is consistent with the “oxidative damage attenuation hypothesis” (Sohal and Weindruch, 1996). Dietary restriction is beneficial in preventing oxidant production, up-regulating antioxidants, and promoting cellular repair systems (Noguera et al., 2011; Ensminger et al., 2021), which may be another reason for the lower oxidative stress and damages levels in high-altitude *N. parkeri*. In addition to temperature and oxygen partial pressure, the sampling site at low altitude was also characterized by more human activities. It has been demonstrated that the production of contaminants and heavy metals in the Tibetan Plateau is closely related to human activities (Bu et al., 2016; Guan et al., 2018). Low elevation habitats typically have higher contaminants and heavy metals, which are potential sources of oxidative stress to frogs (Prokić et al., 2016). Therefore, the higher level of oxidative stress damage exhibited in low-altitude frogs may, at least in part, stem from more contaminants and heavy metals in the lowlands. In addition to damage accumulation resulting from oxidative stress, levels of protein carbonyls and lipid peroxides have also been associated with age in many species (Sohal, 2002). Therefore, it is reasonable that the oxidative damage levels measured in the present study were significantly higher in adults than those in juveniles at both altitudes.

To date, the effect of environmental factors at high altitude on the levels of antioxidant defense mechanisms in ectotherms seems to be controversial. For instance, there was no significant difference in SOD, CAT, GPX, and GST activities between *P. algirus* lizards living at high versus low altitude (Reguera et al., 2014b). However, T-AOC in liver and muscle tissue was significantly lower in high-altitude *P. vlangalii* than that in low-altitude individuals (Zhang et al., 2015). In the present study, the antioxidant defense system showed organ-specific responses to elevated altitude, the activities of SOD, CAT, GPX, and GST, as well as T-AOC, all being significantly higher in the liver of high-altitude adult frogs as compared to low-altitude adults. This is consistent with the fact that liver is the most responsive tissue to abiotic stress and has high constitutive protection against oxidative damage (van der Oost et al., 2003). Other tissues showed fewer responses, GPX activity and T-AOC being significantly increased in heart and brain of high-altitude adults whereas CAT and GPX activities increased significantly in liver of high-altitude juveniles (and T-AOC was also elevated). This organ-specific response may be attributed to different tissues having different oxygen partial pressures and sensitivities to oxygen and ROS (Carreau et al., 2011). The maintenance of high levels of antioxidant defenses in the harsh environment of high altitude (low temperature, hypoxia, and strong UVR) is an activated protective mechanism, which coincides with the phenomenon of “preparation for oxidative stress” (POS) that has received much attention in recent years (Hermes-Lima et al., 2015; Moreira et al., 2016; Moreira et al., 2017). Indeed, preparation for oxidative stress has now been widely reported for many species under different stress conditions, e.g., anoxia/hypoxia, freezing, dehydration, hibernation, estivation, to name a few (Hermes-Lima et al., 2015; Moreira et al., 2016; Moreira et al., 2017; Niu et al., 2021b). For instance, enhanced activity of antioxidant defenses after hypoxia exposure has been reported in many fish, such as Indian catfish, *Clarias batrachus* (Tripathi et al., 2013), common carp, *Cyprinus carpio* (Vig and Nemcsok, 1989), and piapara fish, *Leporinus elongatus* (Wilhelm Filho et al., 2005). Moreover, activities of CAT in muscle and heart, GPX in heart and brain, and GST in brain also increased significantly in leopard frogs (*Rana pipiens*) after 30 h anoxia (Hermes-Lima and Storey, 1996).

High-altitude *N. parkeri* frogs activate and maintain a high level of antioxidant defenses to improve their tolerance to high altitude environments. Increased antioxidant enzyme activities may also arise from enhanced mRNA levels. For instance, the mRNA expression of GPX showed a significant increase in mantle, gill, and hepatopancreas of Pacific oysters (*Crassostrea gigas*) after hypoxia exposure (David et al., 2005). Similarly, in the disk abalone, *Haliotis discus*, hypoxia exposure prompted increased mRNA transcript levels of MnSOD, GPX, and CAT (De Zoysa et al., 2009). Moreover, antioxidant enzymes are regulated by the activation of redox-sensitive transcription factors and post-translational modifications (Ishii et al., 2000; Dawson et al., 2015; Hermes-Lima et al., 2015; Dawson and Storey, 2016). Further studies are needed to determine the regulatory mechanisms employed in changing the antioxidant enzyme activities and antioxidant capacity in *N. parkeri*. Finally, Vc is a non-enzymatic antioxidant and plays a crucial role in protecting cells against the detrimental effects of oxidative stress. In this study, heart Vc content in high-altitude adults and juveniles was significantly higher, as compared with low altitude frogs. A previous study also showed significantly higher levels of Vc in the brains of anoxia-tolerant turtles (*Trachemvs scripta*) than in anoxia-intolerant clawed frogs (*Xenopus laevis*) (Rice et al., 1995), so we speculate that high levels of Vc in high-altitude *N. parkeri* may be closely related to their greater hypoxia tolerance.

## Conclusion

In conclusion, this is the first study to assess hematological parameters and the levels of oxidative stress and antioxidant defenses in *N. parkeri* collected from high and low altitudes. Within the extreme environmental conditions at high altitude, *N. parkeri* can thrive only if they develop physiological and biochemical adaptations to various abiotic stresses. Our findings show that high-altitude juvenile and adult frogs have higher [Hb], Hct, and RBC counts and lower levels of oxidative stress and damage than low-altitude individuals. Both adult and juvenile frogs, *N. parkeri*, exhibit tissue-specific adaptations to environmental stress at high altitude through maintaining higher levels of antioxidant defenses. Future studies are needed to investigate the molecular mechanisms underlying the enhancement of antioxidant defenses. Overall, our present study contributes to elucidating the physiological and biochemical adaptations to high altitude in ectothermic vertebrates.

## Data Availability

The raw data supporting the conclusion of this article will be made available by the authors, without undue reservation.
