# Supplementary material for: Physiological and Biochemical Adaptations to High Altitude in Tibetan Frogs, Nanorana parkeri
Source: Front Physiol. 2022 Jul 6;13:942037. doi: 10.3389/fphys.2022.942037 (PMC9298763; doi:10.3389/fphys.2022.942037)
Supplement: Supplementary file 1 [file DataSheet1.DOCX]

**The principles of the biochemical analyses**

GSH content (product number: A061-1-2) was assayed using a 5,5'-dithiobis-2-nitrobenzoic acid (DTNB)-based cycle reaction and expressed as micromoles per gram of tissue (μmol g^−1^ tissue). MDA is a degradation product of lipid peroxides and was determined using the thiobarbituric acid (TBA) method. MDA condenses with TBA to form a red product with a maximum absorption peak at 532 nm and data are expressed as nanomoles per milligram of protein (nmol mg^−1^ protein) (product number: A003-1-2). Carbonyl derivatives of proteins react with 2,4-dinitrophenylhydrazine (DNPH) to form 2,4-dinitrophenylhydrazone, that can be quantified spectrophotometrically. CG content was expressed as nanomoles per milligram of protein (nmol mg^−1^ protein) (product number: A087-1-2).

Superoxide anion (O_2_^•-^) was produced through the xanthine and xanthine oxidase reaction system, which can oxidize hydroxylamine to form nitrite. This reaction process can be inhibited by SOD and so a decrease in nitrite content is related to SOD activity (product number: A001-1-2). One unit of SOD activity was defined as the amount of SOD per milligram of tissue protein in 1 mL of reaction solution when the SOD inhibition rate reached 50% at 550 nm. CAT can directly decompose its substrate hydrogen peroxide (H_2_O_2_). One unit of activity was deﬁned as the amount of enzyme that consumed 1 μmol of H_2_O_2_ per min at 240 nm (product number: A007-2-1). GPX promotes the reaction of H_2_O_2_ with reduced glutathione (GSH) to form H_2_O and oxidized glutathione (GSSG), and the activity is positively correlated with the consumption of GSH. One unit of GPX activity was deﬁned as the amount required to reduce the GSH concentration by 1 mmol L^-1^ per min per milligram of protein (product number: A005-1-2). GST can catalyze the binding of GSH to 1-chloro-2,4-dinitrobenzene (CDNB, substrate). Its activity is linearly related to the change of substrate concentration within a certain reaction time. One unit of GST activity was deﬁned as the amount required to reduce the GSH concentration by 1 μmol L^-1^ per min per milligram of protein (product number: A004-1-1). GR catalyzes the production of GSH from GSSG in the presence of NADPH-supplied hydrogen and the decrease in absorbance of NADPH at 340 nm is positively correlated with GR activity. One unit of GR activity was defined as the amount required to decrease the concentration of NADPH by 1 mmol L^-1^ per min per milligram of protein at 340 nm (product number: A062-1-1). All antioxidants in tissues can reduce Fe^3+^ to Fe^2+^, the latter can react with phenanthroline to form stable complexes. Hence, total antioxidant capacity (T-AOC) was assayed by the above reaction process and is defined as the amount that made a 0.01 increase in absorbance per min per milligram of protein (product number: A015-1-2). Reduced ascorbic acid (Vc) reacts with Fe^3+^ to form Fe^2+^, which in turn reacts with morpholine to develop color at 536 nm. Vc content was expressed as micrograms per milligram of protein (μg mg^−1^ protein) (product number: A009-1-1).
